# Diagnostic accuracy of Transmitted-light plethysmography for the assessment of pulpal circulation in traumatized young permanent incisors

**DOI:** 10.1038/s41598-025-25063-8

**Published:** 2025-11-28

**Authors:** Satoko Kakino, Hiroaki Ohki, Kaori Kohi, Yuko Matsumura, Tsutomu Iwamoto

**Affiliations:** Department of Pediatric Dentistry/Dentistry for Persons with Special Needs, Division of Oral Health Sciences, Graduate School of Medical and Dental Sciences, Institute of Science Tokyo, 1-5-45 Yushima, Bunkyo-ku, Tokyo, 113-8549 Japan

**Keywords:** Transmitted-light plethysmography (TLP), Cross-correlation analysis, Pulp vitality, Pulpal blood flow, Diagnostic accuracy, Diseases, Health care, Medical research

## Abstract

Transmitted light plethysmography (TLP) is a noninvasive, objective optical method used to assess dental pulp circulation and evaluate pulp vitality. This study aimed to develop a quantitative diagnostic approach for TLP and assess its accuracy through cross-correlation analysis of plethysmographic signals obtained from both the tooth and the finger. A total of 131 maxillary incisors from 97 pediatric patients (aged 6–17 years) were categorized into three groups: nontraumatized teeth, traumatized vital teeth, and traumatized nonvital teeth. Standard diagnostic procedures, including assessments for discoloration, percussion sensitivity, periapical translucency, and electric pulp testing (EPT), were performed alongside TLP evaluation, 3 to 18 months following dental trauma. Tooth plethysmograms were acquired using a 525 nm light-emitting diode directed through the examined teeth, while simultaneous finger plethysmograms were recorded as reference signals. Cross-correlation coefficients (R-values; 0 < *R* < 1) were calculated between the tooth and finger signals for each tooth, and diagnostic cutoff thresholds were determined using receiver operating characteristic (ROC) curve analysis based on the maximum Youden index. Statistically significant differences in R-values were observed among the three groups. The optimal diagnostic cutoff was identified as 0.40 (95% confidence interval: 0.38–0.45), at which TLP demonstrated a sensitivity of 0.94 and a specificity of 0.88, outperforming EPT. Logistic regression analysis incorporating all pulp testing methods revealed a significant association with pulp vitality status. Furthermore, the area under the ROC curve (AUC) for TLP combined with standard tests was significantly greater than that of any individual model. These results indicate that TLP offers improved diagnostic precision for identifying nonvital pulp and may serve as a valuable adjunct to conventional pulp vitality assessments.

**Supplementary Information:**

The online version contains supplementary material available at 10.1038/s41598-025-25063-8.

## Introduction

Conservation of the dental pulp is crucial for maintaining the integrity of traumatized young permanent teeth. Loss of pulp tissue in such teeth can result in complications such as arrested root development and increased susceptibility to root fractures, ultimately compromising long-term prognosis^1^. To mitigate these risks, clinicians should avoid unnecessary pulpectomy procedures. Additionally, in cases of pulp necrosis, timely and accurate diagnosis during the early stages of trauma is essential to prevent the onset and progression of inflammatory root resorption which initially presents as radiographic signs^2^.

The decisions regarding pulpectomy are typically based on a combination of clinical and radiographic findings, including the patient’s pain history, symptom characteristics (e.g. spontaneous or lingering pain), thermal or electric pulp testing, and radiographic signs such as periapical radiolucency. Current guidelines provide further recommendations on treatment selection. Both the European Society of Endodontology (ESE)^3^ and the American Association of Endodontists (AAE)^4^ state that in mature permanent teeth diagnosed with irreversible pulpitis, partial or full pulpotomy may be considered a definitive treatment option. Similarly, the International Association of Dental Traumatology (IADT) guidelines for avulsion^5^ highlight that in immature permanent teeth with open apices, spontaneous pulp revascularization may occur, and root canal treatment should be avoided unless clinical or radiographic signs of pulp necrosis or infection are evident on follow-up. These guidelines emphasize that case selection, pulpal status, and root maturity must be carefully considered when determining the most appropriate treatment approach.

However, conventional methods for assessing pulp vitality, such as thermal and electric pulp tests, present limitations in clinical practice. These tests depend on subjective patient responses^6^, which can compromise diagnostic accuracy, especially in pediatric patients^7^. Moreover, false-positive results are common owing to elevated pulpal nerve thresholds in recently erupted young permanent teeth or teeth affected by early-stage trauma^8–12^. Consequently, there is a pressing need to develop a reliable, objective method for diagnosing pulp vitality, particularly in young permanent teeth.

Transmitted-light plethysmography (TLP) is a noninvasive, objective technique used to assess blood flow in the dental pulp. Several prior studies have reported the application of photoplethysmography in human teeth^13–19^. In addition, more recent in vitro studies using extracted molars^20,21^ have suggested the potential clinical applicability of photoplethysmography. TLP pulse waveforms reflect cyclic changes in blood volume within the microvascular bed, synchronized with the cardiac cycle. This method enables the detection of pulpal blood flow in traumatized deciduous or young permanent teeth, which may not respond to neuronal stimulation^10,16,18^. In our study, a 525-nm light-emitting diode (LED) was used to capture transmitted light signals through the pulp cavity of the incisors^17,22,23^, while finger plethysmography was concurrently employed as a heartbeat reference. Previous observations indicated that TLP pulse waveforms gradually change following dental trauma, depending on the clinical prognosis of the pulp. When pulp tissue is damaged, the waveform becomes temporarily indistinct, with TLP amplitude decreasing over time and ultimately disappearing as necrosis sets in ^16,18,19^. However, such visual assessments are inherently subjective and insufficient for precisely evaluating pulpal blood flow or distinguishing between vital and nonvital tissue. Therefore, a quantitative approach to TLP assessment is necessary for broader clinical application.

In this study, we employed the cross-correlation coefficient between tooth and finger plethysmograms as a quantitative indicator of relative pulpal blood circulation. Cross-correlation analysis is a technique used to assess the similarity between time-dependent signals and is typically applied in medical engineering to explore relationships between various biomedical signals^24–27^. One advantage of applying cross-correlation analysis to pulp diagnosis is that the resulting coefficients are independent of differences in transmitted light intensity caused by anatomical variations among individual teeth. We hypothesized that the degree of cross-correlation between tooth and finger plethysmograms could serve as a reliable measure of pulpal circulation, enabling the objective differentiation between vital and nonvital teeth.

To our knowledge, no prior studies have quantitatively assessed the diagnostic accuracy of tooth plethysmography for evaluating pulp vitality. Therefore, the objective of this study was to develop a novel diagnostic approach for TLP based on cross-correlation analysis between tooth and finger plethysmograms to quantitatively assess pulpal blood flow. In addition, we evaluated and compared the diagnostic performance of TLP and conventional pulp vitality tests in distinguishing vital from nonvital teeth, thereby examining the clinical utility of TLP in pulpal diagnosis.

## Materials and methods

### Selection of subjects and teeth

This study was approved by the Ethics Committee of the Graduate School of the Institute of Science Tokyo (Approval No. D2016-063). All methods were carried out in accordance with relevant guidelines and regulations, including the Declaration of Helsinki and the Ethical Guidelines for Medical and Health Research Involving Human Subjects in Japan. Seventy-five patients who visited the pediatric dental clinic at the Institute of Science Tokyo Hospital between 2013 and 2023 were considered for inclusion. All patients presented with dental trauma involving the maxillary central or lateral permanent incisors. Informed consent was obtained from all participants and their guardians after providing a detailed explanation of the study’s purpose and methodology.

The subjects’ teeth were categorized into three groups: nontraumatized (Group 1, *n* = 37), traumatized vital (Group 2, *n* = 62), and traumatized nonvital (Group 3, *n* = 32). For Group 1, 37 nontraumatized maxillary incisors were selected from 37 children (mean age: 11.3 years; range: 7–17 years; male: *n* = 15; female: *n* = 22). All participants in this group were healthy volunteers whose guardians provided consent for participation. Inclusion criteria for Group 1 were: (1) clinically healthy maxillary central incisors with no dental caries, restorations, periodontal disease, history of orthodontic treatment, or prior dental trauma; (2) no radiographic evidence of periapical radiolucency, root resorption, inflammation, or pulp canal obliteration; and (3) confirmed pulp sensibility using an electric pulp test. Radiographic examinations were conducted for all Group 1 subjects.

Inclusion criteria for traumatized teeth and patients (Groups 2 and 3) were: (1) absence of systemic disease and no history of long-term medication use; (2) cooperative behavior during all pulp vitality tests; and (3) an observation period of 3 to 18 months following the traumatic event. Among these cases, 94 teeth from 60 patients (mean age: 9.3 years; range: 7–16 years; male: *n* = 38; female: *n* = 22) fulfilled the inclusion criteria. In Group 3, 32 teeth were measured and included in the analysis.

**Fig. 1:**
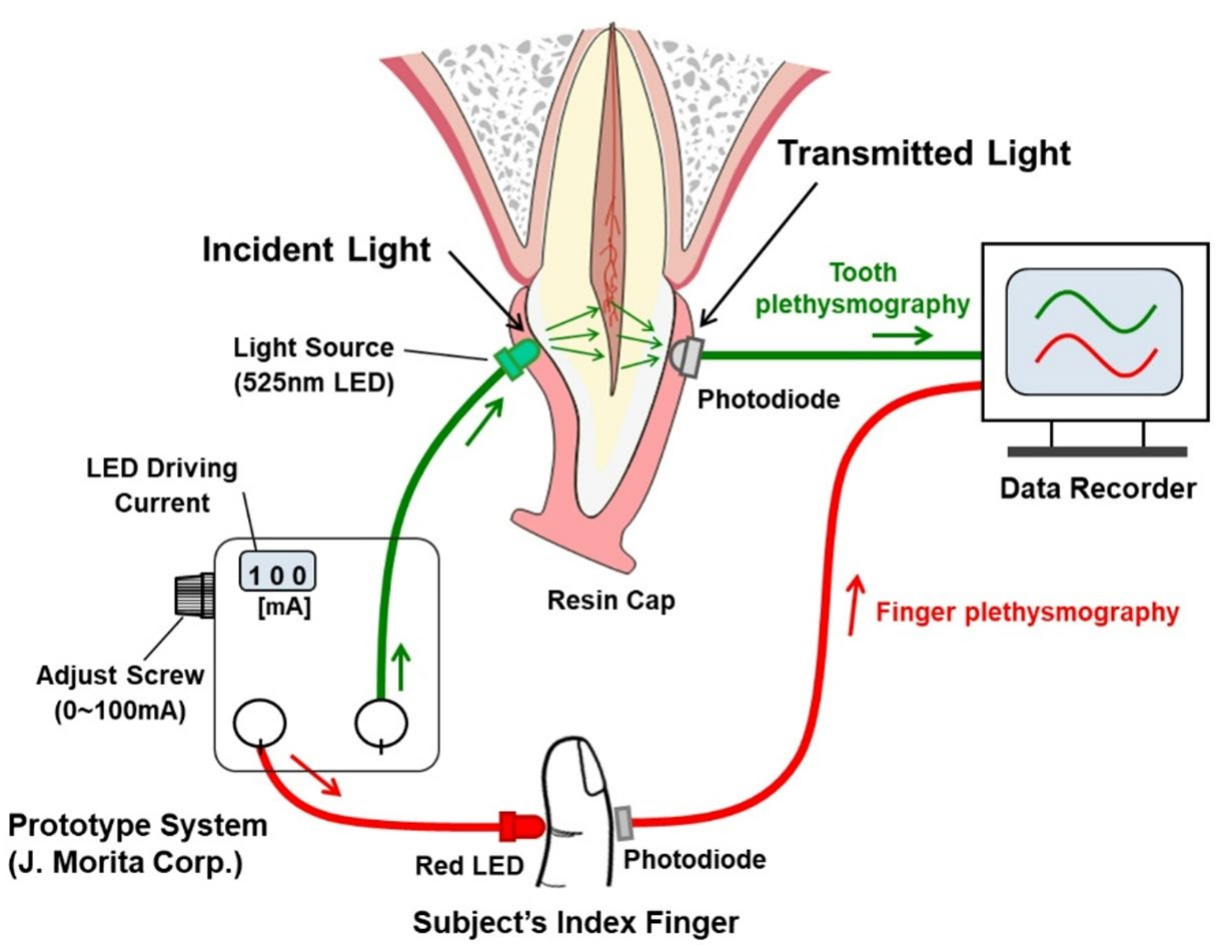
Schematic of the TLP system. A TLP system with a 525-nm green LED was used to detect pulpal blood flow in the maxillary incisors. A typical Si-PIN photodiode was used as the photodetector. A finger plethysmogram was simultaneously recorded as the reference signal. An individual acrylic resin cap was prepared for the TLP measurement of each tooth.

The target sample size of 96 subject teeth was determined based on sample size estimation for diagnostic test studies^28^, assuming a predetermined sensitivity (*P*_0_) of 85% and an expected sensitivity (*P*_1_) of 95%, with 90% power to detect the difference (*P*_1_ − *P*_0_) at a 95% confidence level.

### Standard diagnostic pulp tests for traumatized teeth

Traumatized teeth were classified according to standard categories of dental trauma, including subluxation, luxation, intrusion, extrusion, avulsion, crown fracture, and root fracture^29^. Each subject tooth underwent evaluation using commonly accepted pulp diagnosis and sensibility tests, including assessment of coronal discoloration, percussion response, radiographic apical translucency, the electric pulp test (EPT), and transmitted light plethysmography (TLP). All diagnostic procedures were conducted at the Pediatric Dental Department of the Institute of Science Tokyo Hospital by licensed dentists with more than eight years of clinical experience in pediatric dentistry. Coronal discoloration was assessed through visual inspection under light transillumination. EPT was carried out using the Sybron Endo Vitality Scanner™ (model 2006, Sybron Endo Dental Specialties, Glendora, CA, USA), and each tooth was tested twice, with the results compared to those of the contralateral control tooth. To evaluate the presence or absence of apical periodontitis, radiographic imaging was performed and analyzed using the Periapical Index (PAI) scoring system^30^. The PAI assesses apical translucency on a scale from 1 to 5, where: (1) denotes normal periapical structure, (2) indicates small changes in bone structure, (3) reflects bone structure changes with some mineral loss, (4) signifies periodontitis with a well-defined radiolucency, and (5) represents severe periodontitis with exacerbating features. PAI scores of 3 to 5 were considered indicative of apical lesions. In addition, the stage of root development was determined according to the Moorrees classification of root formation^31^.

For traumatized nonvital teeth in Group 3, the reference standard for classification was based on multiple findings from standard pulp diagnostic tests. These included radiographic evidence of periapical radiolucency, percussion sensitivity, responses to sensibility tests such as electric pulp testing (EPT), among others. Teeth with periapical radiolucency were classified as nonvital, while in cases without lesions, persistent EPT nonresponse or marked percussion pain supported the diagnosis. When findings remained equivocal, a test cavity was prepared, and absence of pulpal bleeding confirmed pulp necrosis.

### TLP measurement

TLP measurements were performed in the examination room adjacent to the pediatric dental department, and the data were analyzed by two pediatric dentists with over 10 years of experience in conducting TLP. The measurements were taken either on the same day as the standard pulp tests or within two weeks thereafter. Before TLP recording, the surface of the subject tooth was polished to remove any external stains that might interfere with light transmission. A schematic representation of the TLP system is shown in Fig. 1. The TLP device, equipped with a 525-nm green LED (prototype system, J Morita Corp., Kyoto, Japan), was used to detect pulpal blood flow in the maxillary incisors. The 525-nm wavelength was chosen for its high sensitivity to blood volume changes in upper incisors^17,19,29^. A silicon PIN photodiode (spectral sensitivity: 450–1110 nm, peak sensitivity at 920 nm; HPS 304AI, Kodenshi Corp., Tokyo, Japan) was used as the photodetector. A finger plethysmogram was recorded simultaneously as a reference signal (TL-631T3; Nihon Kohden Corporation, Tokyo, Japan).

For each subject tooth, an individual acrylic resin cap was fabricated for the TLP measurement. Two openings were created in the cap to accommodate the 3-mm green LED (OSPG 3131P, Optosupply International) on the palatal surface and the photodiode on the labial surface. During the procedure, subjects were placed in a supine position. The TLP measurement for each tooth required approximately 90 s. All signal data were recorded using a data acquisition system (LabChart, AD Instruments Pty. Ltd., Australia).

A cross-correlation analysis between the tooth and finger plethysmograms of all subject teeth was conducted using the Complex System Chaos Analysis Program (TAOS Institute, Inc., Yokohama, Japan). Cross-correlation is a commonly used method for quantifying the similarity between two time-series signals, allowing for the assessment of temporal relationships and signal alignment. The calculation formula is as follows:

$$\:R\left(m\right)=\frac{1}{N}\sum\:_{n=0}^{N-1}x\left[n\right]y\left[n+m\right]\:\:\:(m=0,\:1,\:2,\dots\:(N-1))$$,

where x[n] is a sampled function of x(t), that is, a function of the TLP waveform, and y[n] is a function of y(t), that is, a function of the finger plethysmogram waveform, where N is the number of samplings.

We utilized the absolute value of the cross-correlation coefficient (R), which ranges from 0.0 to 1.0. An R value approaching 1.0 indicates a high degree of similarity between the two time-series signals, suggesting that the tooth plethysmograms were clearly detected and synchronized with the reference finger plethysmograms. Conversely, an R value near 0.0 reflects low similarity, indicating an absence or significant reduction of detectable pulpal circulation. For each tooth, 45 TLP pulse waveforms were analyzed to ensure consistent and reliable signal evaluation.

### Statistical analysis

Since the cross-correlation coefficients in Group1 and Group3 did not follow a normal distribution, nonparametric statistical methods were required. Consequently, intergroup comparisons of cross-correlation coefficients among Groups 1–3 were analyzed using the Kruskal–Wallis test, followed by post hoc comparisons with the Mann–Whitney test. Radiographic images were independently assessed by two pediatric dentists to assign a Periapical Index (PAI) score ranging from 1 to 5. Inter-evaluator reliability was assessed using Cohen’s kappa coefficient. The optimal cutoff value of the TLP test for identifying pulp vitality was determined through receiver operating characteristic (ROC) curve analysis based on the maximum Youden index, which indicated that an R value ≤ 0.40 represented the optimal threshold.

To identify potential diagnostic predictors of pulp vitality, univariate logistic regression analysis was performed for each pulp test, with odds ratios and 95% confidence intervals (CIs) calculated. Multicollinearity among all the diagnostic predictors were also assessed. In the logistic regression model, teeth with vital pulp (Groups 1 and 2) were coded as 0, and those with nonvital pulp (Group 3) as 1. The binary outcome was predicted based on the results of the individual pulp tests. Sensitivity, specificity, positive predictive value (PPV), and negative predictive value (NPV) were also calculated for each diagnostic method. To compare the diagnostic performance of TLP alone versus the combined use of standard pulp tests, ROC curves were generated. Areas under the ROC curve (AUCs) with 95% CIs were obtained using multivariate logistic regression analysis. To evaluate the generalizability of the logistic regression model, internal validation was conducted using bootstrapping, and the AUC and its 95% CIs were derived from the resampled data. Statistical significance was defined as *P* < 0.05. All analyses were conducted using STATA software (version 18.0, StataCorp LLC, College Station, TX, USA), except for the estimation and internal validation of the optimal cutoff value, which were performed using R software (version 4.4.2., R Foundation for Statistical Computing, Vienna, Austria) with bootstrapping to obtain the cutoff value and its 95% CI.

## Results

Table 1 presents the characteristics of the subject teeth included in this study. A total of 37 teeth were classified as nontraumatized (Group 1). Within Group 2, 46.8% of the teeth were categorized as having experienced luxation. In Group 3, a notable proportion of dislocation-related injuries was observed, including 25.0% avulsions, 21.9% luxations, 18.8% luxations accompanied by crown fractures, and 12.5% intrusions.

**Table 1:** Group classification and types of dental trauma for young permanent incisors (*N* = 131 teeth from 97 pediatric patients).

	Group 1 Nontraumatized teeth (*N* = 37)		Group 2 Traumatized vital teeth (*N* = 62)		Group 3 Traumatized nonvital teeth (*N* = 32)	
	N	%	N	%	N	%
No trauma	37	100.0%	0	0.0%	0	0.0%
Subluxation	0	0.0%	12	19%	2	6.3%
Subluxation + Crown fracture	0	0.0%	4	6.5%	0	0.0%
Luxation	0	0.0%	29	46.8%	7	21.9%
Luxation + Crown fracture	0	0.0%	2	3.2%	6	18.8%
Intrusion	0	0.0%	2	3.2%	4	12.5%
Intrusion + Crown fracture	0	0.0%	0	0.0%	1	3.1%
Extrusion	0	0.0%	0	0.0%	0	0.0%
Avulsion	0	0.0%	1	1.6%	8	25.0%
Crown fracture	0	0.0%	10	16.1%	4	12.5%
Root fracture	0	0.0%	2	3.2%	0	0.0%

**Fig. 2:**
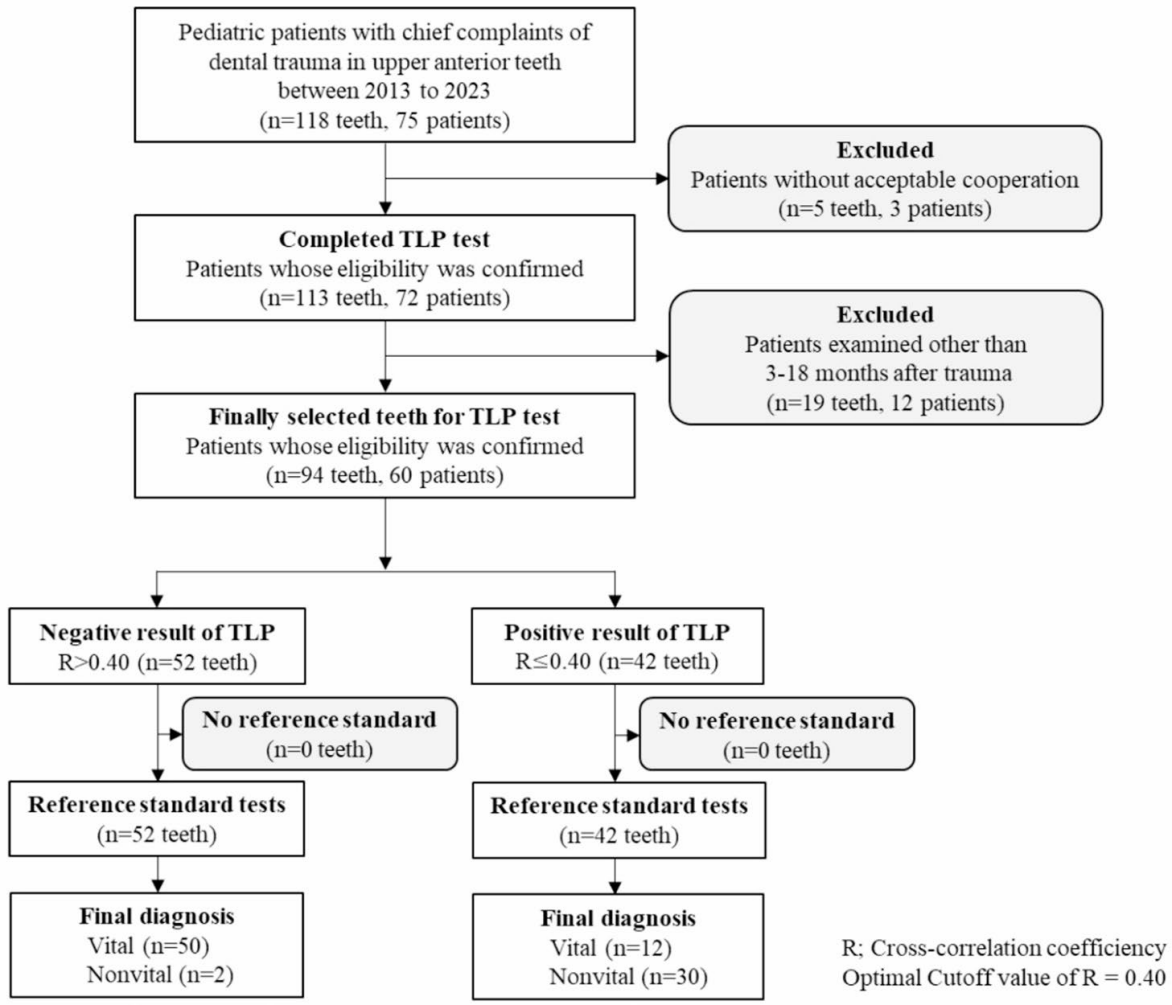
Flow diagram of the patients and traumatized teeth. Seventy five patients who visited the pediatric dental clinic from 2013 to 2023 were potentially eligible for inclusion in this study. Their chief complaints were dental trauma in the upper central or lateral permanent incisors. The reference standard tests were performed for all teeth selected for the TLP test, and the final diagnoses by both TLP and reference standard tests are presented.

Figure 2 illustrates the flow diagram outlining the patient selection process and the distribution of traumatized teeth (Groups 2 and 3). All teeth selected for TLP measurement underwent the reference standard pulp tests, and the final diagnoses based on both TLP and reference standards are shown. A negative TLP result indicates successful detection of pulse waves with an R value greater than the cutoff value of 0.40. A positive TLP result indicates the absence of detectable pulse waves, with an R value of 0.40 or less.

According to the Moorrees classification of root development stages, the distribution of traumatized teeth in Groups 2 and 3 was nearly equal: 53% were in the open-apex stages (R1/2–R3/4), while 47% were in the completed-root stages (Rc, A1/2, and Ac) (*Supplementary Table*). In Group 3, 25% of the teeth were suspected to have arrested root development.

**Fig. 3:**
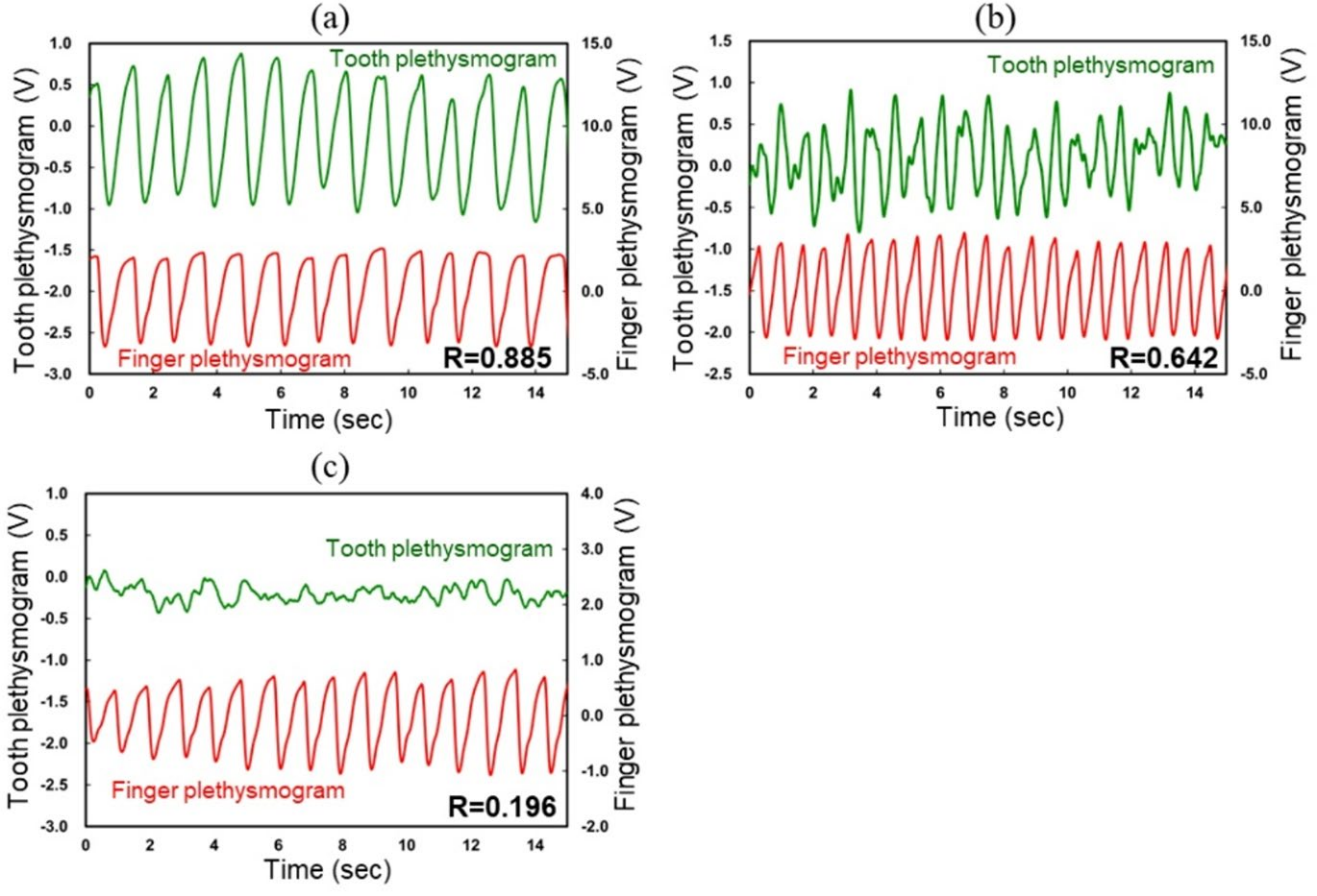
Representative TLP recordings of each group. Group 1: Nontraumatized teeth. Male patient of 14 years and 3 months of age. Group 2: Traumatized vital teeth. Female patient aged 9 years and 0 months with crown fracture. Group 3: Traumatized nonvital teeth. Male patient aged 11 years and 11 months with crown fracture. R: Cross-correlation coefficient.

Figure 3 shows representative TLP recordings from each group. In nontraumatized teeth (Group 1; Fig. 3a), the TLP signal showed distinct pulse waves with large amplitudes that were clearly synchronized with the finger plethysmogram. In traumatized vital teeth (Group 2; Fig. 3b), TLP signals showed lower amplitudes, and pulse shapes were occasionally unclear. In cases of severe trauma within Group 2, TLP pulse waves sometimes disappeared altogether. In nonvital teeth (Group 3; Fig. 3c), TLP waveforms were consistently unclear, with markedly reduced amplitudes and no apparent synchronization with the reference finger plethysmogram.

**Fig. 4:**
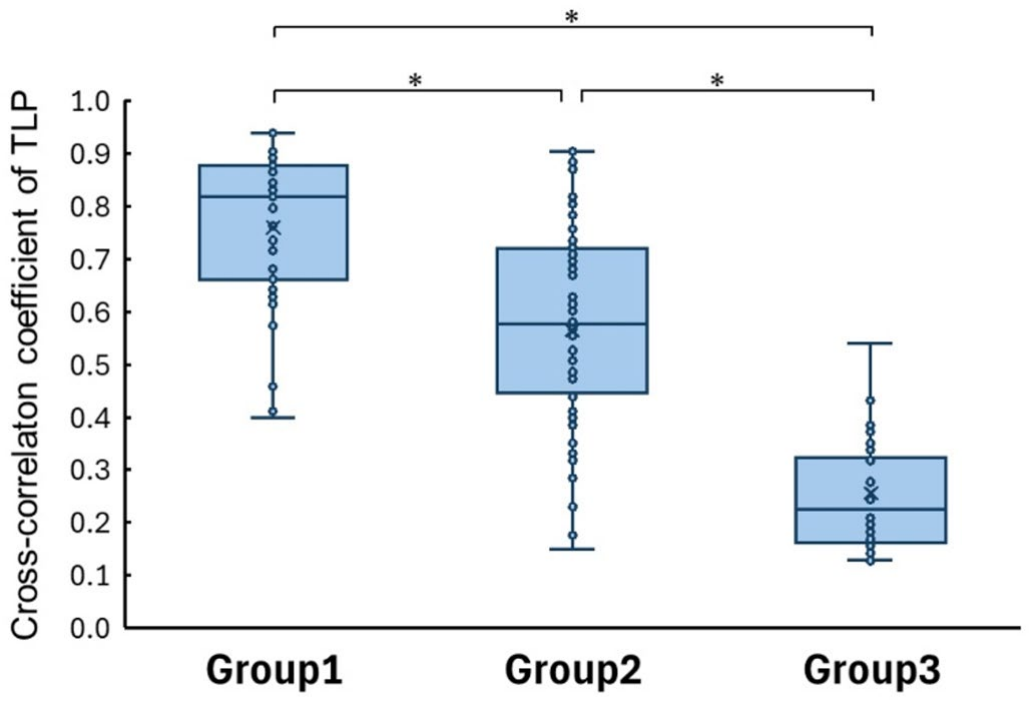
Distribution and intergroup comparison of TLP cross-correlation coefficients. Box plot representing the distribution of TLP cross correlation coefficients. Group1, Nontraumatized teeth; Group2, Traumatized vital teeth; Group3, Traumatized nonvital teeth. The statistically significant differences were observed among the three groups (* indicates *P* < 0.001). The median and interquartile range (IQR) of the cross-correlation coefficient for TLP were 0.818 (IQR: 0.662−0.878) in Group1, 0.578 (IQR: 0.446−0.721) in Group2, and 0.226 (IQR: 0.162−0.324) in Group 3.

As shown in Fig. 4, statistically significant differences in cross-correlation coefficients were observed among the three groups (*p* < 0.001). The median and interquartile range (IQR) of the TLP cross-correlation coefficient were 0.818 (IQR: 0.662–0.878) in Group 1, 0.578 (IQR: 0.446–0.721) in Group 2, and 0.226 (IQR: 0.162–0.324) in Group 3.

Table 2 presents the results of the standard pulp tests for nontraumatized teeth (Group 1), traumatized vital teeth (Group 2), and traumatized nonvital teeth (Group 3). For the evaluation of periapical translucency using the Periapical Index (PAI), the inter-evaluator agreement, as measured by Cohen’s kappa coefficient, was 0.81, indicating strong reliability. The prevalence of negative responses to EPT was 5.4% in Group 1, 16.1% in Group 2, and 71.9% in Group 3. The proportion of teeth with TLP results showing *R* ≤ 0.40 was 0.0% in Group 1, 19.4% in Group 2, and 93.8% in Group 3. In Group 2, periapical radiolucency was observed in four of 62 teeth, but findings from other standard pulp diagnostic tests were unremarkable, and the teeth were not diagnosed as necrotic pulp. In Group 3, periapical radiolucency was present in 26 of 32 teeth. Among the remaining six, five showed percussion pain and no EPT response, and one showed progressive crown discoloration with persistent lack of EPT response. In all six teeth, cavity tests were performed, and no pulpal bleeding was observed, confirming pulp necrosis.

Table 3 summarizes the odds ratios and 95% confidence intervals derived from univariate logistic regression analyses of each pulp test. Sensitivity, specificity, positive predictive value (PPV), and negative predictive value (NPV) are also reported. All pulp tests demonstrated a statistically significant association with pulp vitality. Multicollinearity was not detected among the diagnostic predictors. The highest odds ratio was observed for TLP, followed by apical radiolucency. Among all tests, TLP exhibited the highest sensitivity and NPV.

**Table 2:** Pulp tests for the diagnosis of nontraumatized and traumatized teeth. EPT, Electric pulp test; TLP, Transmitted-light plethysmography (N=131 teeth from 97 pediatric patients).

	Group 1 Nontraumatized teeth (*N* = 37)		Group 2 Traumatized vital teeth (*N* = 62)		Group 3 Traumatized nonvital teeth (*N* = 32)	
	N	%	N	%	N	%
Discoloration	0	0.0%	3	4.8%	6	18.8%
Percussion	0	0.0%	4	6.5%	17	53.1%
Periapical translucency	0	0.0%	5	8.1%	26	81.3%
EPT (-)	2	5.4%	10	16.1%	23	71.9%
TLP (R ≤ cut off value = 0.40)	0	0.0%	12	19.4%	30	93.8%

**Table 3:** Univariate logistic regression analysis of each pulp test. CI, confidence Interval; EPT, electric pulp test; TLP, Transmitted-light plethysmograpy; PPV, positive prediction; NPV, negative prediction.

	Odds Ratio	[95% CI]	*p* value	Sensitivity	Specificity	PPV	NPV
Discoloration	7.38	1.73–31.55	0.007	0.19	0.97	0.67	0.79
Percussion	26.72	7.96–90.97	< 0.001	0.53	0.96	0.81	0.86
Periapical translucency	81.47	23.02–288.29	< 0.001	0.81	0.95	0.84	0.94
EPT (-)	16.91	6.43–44.44	< 0.001	0.72	0.87	0.64	0.91
TLP (R ≤ cut off value = 0.40)	108.75	23.00–514.12	< 0.001	0.94	0.88	0.71	0.98

**Fig. 5:**
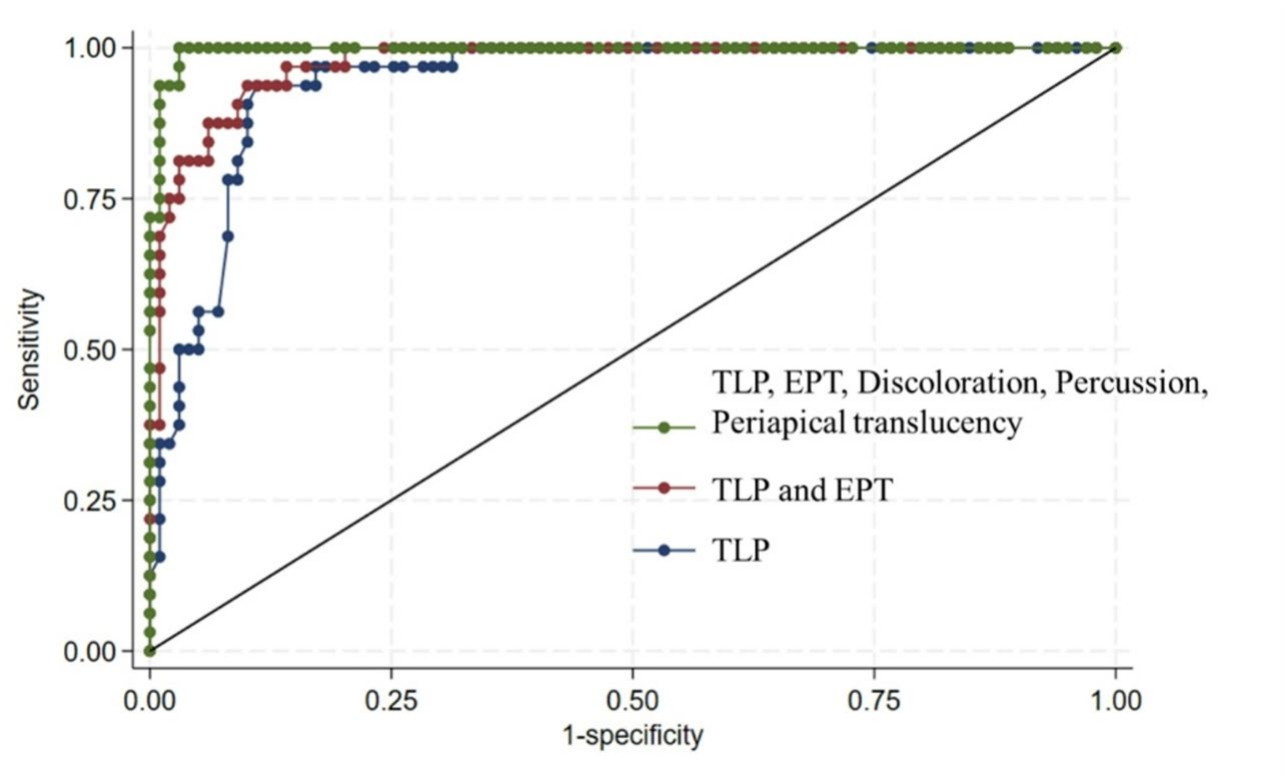
Receiver operating characteristic (ROC) curves predicted based on univariate and multivariate logistic regression models. (a) TLP. (b) TLP and EPT. (c) TLP, EPT, Discoloration, Percussion, Periapical translucency. The AUC of the model with TLP and standard pulp tests showed the highest AUC values compared to the models with TLP alone or TLP in combination with EPT. There was a statistically significant difference in the AUC among the three logistic regression models (*p* < 0.05). TLP, transmitted-light plethysmography; EPT, electric pulp test; ROC, receiver operating characteristic.

Figure 5 presents the ROC curves generated from the logistic regression models. The ability to differentiate pulp vitality was enhanced in the model that integrated multiple pulp tests. The area under the curve (AUC) for the model combining TLP with standard pulp tests was the highest, surpassing the AUCs of models using TLP alone or TLP with EPT. The estimated AUCs (with 95% CIs), obtained through internal validation, were 0.941 (0.894–0.974) for TLP alone, 0.973 (0.938–0.990) for TLP in combination with EPT, and 0.996 (0.984–1.000) for TLP with all standard pulp tests. There was a statistically significant difference in the AUC among the three logistic regression models (*p* < 0.05). The corresponding optimal diagnostic cutoff value, internally validated via bootstrapping, was 0.40 (95% CI: 0.38–0.45).

## Discussion

The results of this study demonstrate that pulp vitality can be quantitatively assessed using cross-correlation analysis of tooth and finger plethysmographic signals. The findings further indicate that the diagnostic accuracy of TLP, when combined with other pulp tests, exhibits superior sensitivity compared to conventional standard diagnostic methods.

The cross-correlation coefficient (R-value) varied according to pulp vitality status. As illustrated in the representative plethysmographic recordings (Fig. 3), Group 1 exhibited higher R-values, corresponding to well-defined TLP waveforms with large amplitudes clearly synchronized with the finger plethysmogram. By contrast, Group 2 showed reduced R-values owing to unstable waveforms with diminished amplitudes, consistent with previous findings^16,18,19^. Group 3 exhibited the lowest R-values, reflecting an absence or significant reduction in synchronization with the reference signal and markedly diminished amplitudes. Clinical confirmation during access cavity procedures revealed an absence or minimal presence of pulpal blood in all Group 3 teeth, consistent with a nonvital status. Variations in hard tissue thickness and age-related optical properties can influence transmitted-light intensity^10,16^, however, as the pulp undergoes necrosis, the pulpal signal amplitude diminishes regardless of theses anatomical or optical differences. As described, the cross-correlation analysis enabled a quantitative evaluation of relative pulpal blood flow, providing an objective metric for diagnosing pulp vitality.

This study employed cross-correlation analysis to quantify the detectability of TLP pulse waves and, for the first time, validated the diagnostic accuracy of TLP in pulp vitality assessment. Cross-correlation analysis has been widely utilized for the quantitative evaluation of biological activities, with prior applications in assessing cardiovascular oscillation, respiration, enterokinesis, and cerebral hemodynamics^24–27^. However, no previous studies have applied this method to the diagnosis of dental pulp vitality. Given that the R-value in TLP is a continuous variable (0 < *R* < 1), a diagnostic cutoff value was established to differentiate the presence or absence of pulpal blood flow—effectively distinguishing vital from non-vital pulp. The optimal cutoff value for R was determined to be 0.40, with high sensitivity and specificity values of 0.94 and 0.88, respectively.

In this study, TLP exhibited the highest sensitivity among the reference tests, highlighting its potential superiority in diagnosing nonvital pulp conditions such as pulp necrosis. Electric pulp testing (EPT) was used as the sensibility test owing to its capacity to deliver standardized stimulation to the dental pulp. According to recent systematic reviews on pulp vitality testing^32–36^, the reported sensitivities for EPT, laser Doppler flowmetry (LDF), and pulse oximetry (PO) range from 0.63 to 0.87, 0.82–1.0, and 0.81–0.97, respectively, with corresponding specificities of 0.74–1.0, 0.95–1.0, and 0.95–0.98. These findings suggest that EPT, which typically demonstrates lower sensitivity and higher specificity, may be more reliable for identifying vital pulp rather than nonvital pulp.

Consistent with previous reports, the sensitivity of EPT in this study was lower than that of TLP. One contributing factor to false-negative results with EPT is its ability to provoke a response even in nonvital teeth, owing to the resilience of the pulp nerves to inflammatory conditions^2,36–38^. In our findings, EPT produced responses in 9 of 32 nonvital teeth (Group 3), and among those 9 teeth, pulpal blood flow was undetectable by TLP in 8 cases. This outcome supports the diagnostic accuracy of TLP in correctly classifying nonvital teeth. Conversely, some false-positive outcomes were also observed. In Group 1 (nontraumatized young permanent teeth) and during the early stages of trauma in Group 2, transient increases in pulpal sensitivity threshold led to absent EPT responses, consequently lowering the specificity of EPT. These results suggest that TLP may address the diagnostic limitations associated with EPT.

Although blood flow contamination from surrounding periodontal tissues has been recognized as a potential source of false-negative results—specifically, the erroneous detection of pulpal blood flow in nonvital teeth^39^—our TLP system, which employs a 525-nm LED, has previously been validated through in vivo studies and theoretical modeling using Monte Carlo simulations^17^. These investigations confirmed that soft tissue interference had a negligible impact on the measurement outcome, thereby contributing to the high sensitivity observed in this study.

By contrast, the specificity of TLP was lower than that of the reference pulp test. False-positive TLP results, defined as the inability to detect pulpal blood flow in teeth that were clinically diagnosed as vital, occurred in several severe trauma cases within Group 2. In these instances, TLP failed to capture clear plethysmographic signals in teeth exhibiting advanced pulp canal obliteration or pronounced crown discoloration. Moreover, TLP signal of certain severe trauma cases may be diminished during the healing process and occasionally fail to reach the cutoff value. Importantly, however, clinical decisions are not based on TLP findings alone; additional diagnostic information such as periapical radiographs, percussion, and electric pulp testing is also considered. Thus, even when TLP signals are weak, endodontic treatment is not initiated unless pulp necrosis is confirmed, which helps to avoid over-diagnosis and unnecessary interventions.

The results for positive predictive value (PPV) and negative predictive value (NPV) offer a meaningful characterization of the diagnostic performance of TLP. PPV reflects the proportion of true nonvital teeth among those classified as positive by TLP, whereas NPV indicates the proportion of true vital teeth among those classified as negative. In this study, the NPV of TLP was 0.98, which surpassed that of conventional diagnostic methods. As previously discussed, this high NPV is likely due to the low occurrence of false negatives—instances where a pulse was detected in a clinically non-vital tooth—highlighting the minimal influence of periodontal tissue contamination on TLP measurements.

By contrast, the PPV of TLP was 0.71, which is relatively lower than that of some standard diagnostic tests. This reduced PPV can be attributed to weak TLP pulse signals observed in certain cases within Group 2, particularly in severely traumatized teeth or those in the early phases of healing. Despite the weak plethysmographic signals, these teeth were determined to be vital and did not require intervention based on comprehensive clinical evaluations. These findings suggest that TLP signals may still accurately reflect the underlying pathological state of traumatized teeth, even when intervention is not indicated.

Our logistic regression analysis (Fig. 5) revealed statistically significant differences in the area under the curve (AUC) values among models using TLP alone, TLP combined with EPT, and all reference tests. These results support the notion that combining multiple diagnostic tools enhances overall diagnostic accuracy. Previous studies have similarly reported improved accuracy through the integration of EPT with pulse oximetry (PO), laser Doppler flowmetry (LDF), or thermal testing^35^. A recent systematic review identified the absence of a definitive clinical reference standard as a major limitation in pulp diagnostics, highlighting the need for a multifactorial diagnostic framework^40^. Given that each diagnostic method possesses unique strengths and limitations, a comprehensive strategy that incorporates various clinical tests is essential for achieving accurate and reliable pulp vitality assessments.

In previous studies, laser Doppler flowmetry (LDF) and pulse oximetry (PO) have been reported as diagnostic methods for assessing pulp vitality based on pulpal blood flow measurements^41–44^. These techniques enable the evaluation of microcirculation within the dental pulp using commercially available equipment, although the interpretation of their results has varied across studies. The first report of pulpal blood flow measurement in humans using LDF was published by Gazelius et al.^41^. Subsequently, Evans et al.^42^ proposed a cutoff value of 7.0 perfusion units (PU) for determining pulp vitality. Other studies have introduced alternative criteria, such as defining a tooth as vital if the ratio of diseased pulp flux to healthy pulp flux is ≥ 0.6^43,44^.

Numerous studies have reported the effectiveness of pulse oximetry (PO) as a diagnostic tool for assessing pulp vitality^2,45,46^. PO typically estimates the oxygen saturation (SO_2_) levels in pulpal blood by analyzing pulsatile changes detected via dual-wavelength plethysmography, using near-infrared and red light sources. Previous investigation have performed PO measurements during the early stages following dental trauma (0 days to 6 months), applying diagnostic criteria from earlier studies that defined vital pulp as having SO_2_ values in the range of 72%–82%^2^. However, the absolute quantification of SO_2_ in the dental pulp has proven challenging, as highlighted by in vitro studies^14^. The small size of the pulp chamber and its encasement within mineralized tissue limit the effectiveness of longer-wavelength light. Consequently, visible light with shorter wavelengths is more suitable for optical assessment of dental tissues than red or near-infrared light^14,39^. These observations align with our previous findings on the use of plethysmography across multiple wavelengths and studies of the optical properties of dental structures^17,22,23^.

TLP offers distinct advantages over other methods of pulpal blood flow measurement, primarily owing to its use of an LED light source that is affordable, compact, and safe^19^. Additionally, previous studies have shown that the effect of light scattering on the enamel surface is less pronounced in TLP than in reflective LDF measurements^47,48^. The 525 nm wavelength employed in our TLP system is particularly effective, as it is strongly absorbed by hemoglobin within the pulp cavity. This characteristic allows for high detection sensitivity in tooth plethysmography, surpassing that of red and near-infrared wavelengths commonly used in other optical methods^17,22,23^.

Despite the advantages of TLP, clinical and technical limitations include situations where the method may provide unreliable results, such as teeth with pulp canal obliteration due to trauma^1^. In such cases, narrowing the pulp chamber and increased dentin thickness can attenuate detectable blood flow signals and occasionally lead to false positive (nonvital) results. Another practical issue is the use of customized resin caps to stabilize the LED and photodiode, which minimizes operator variability but is time-consuming and impractical for routine clinical use. Furthermore, as the present measurements were performed using a prototype device, which limited direct clinical extrapolation, further refinements and standardization will be necessary to facilitate routine application. In addition, while previous in vivo^14,39^ and in vitro studies^14,20,21,23^ have investigated the use of TLP in posterior teeth, differences in tooth structure suggest that optimization of probe design and wavelength selection, as well as the development of universal probes, will be necessary for broader applicability. Moreover, because this study included only pediatric anterior teeth, the generalizability of the findings is restricted. However, given the known differences in optical transmission between young and adult permanent teeth^10^, further validation in adult teeth will provide important evidence for enhanced clinical use.

As for the statistical aspects, only internal validation using bootstrapping was performed in this study. While this approach reduces potential optimism in the estimated performance of the models, it cannot fully exclude optimism bias. External validation employing independent datasets will be required to confirm the generalizability of our findings.

The current study was designed as a cross-sectional investigation, focusing on pulp testing in teeth examined within 3 to 18 months following traumatic injury. Immediate post-trauma assessments were excluded for two primary reasons. First, periapical radiolucency observed shortly after trauma may reflect transient bone remodeling rather than true pulpal pathology, thereby compromising the reliability of radiographic diagnosis^49,50^. Second, the sensitivity threshold in pulp sensibility tests such as EPT can be temporarily elevated in the early stages of trauma, resulting in false-positive responses owing to transient nonresponsiveness of the pulpal nerve^1,6–10^. Andreasen et al. reported that pulp sensation in young permanent teeth typically begins to recover within two months post-trauma^9^. One of the important advantages of TLP is its ability to objectively assess pulp vitality even in the early stages after trauma.

In conclusion, the current study demonstrated that TLP can serve as an effective tool for the quantitative evaluation of pulpal blood circulation through cross-correlation analysis between tooth and finger plethysmographic signals. The TLP test exhibited higher sensitivity than the reference pulp test, indicating improved diagnostic accuracy in detecting nonvital pulp. These results highlight the potential utility of TLP as a complementary method alongside standard pulp diagnostic tests.

Future research will focus on longitudinal assessments of traumatized teeth using cross-correlation analysis to investigate the relationship between pulpal blood flow recovery and pulp vitality. Furthermore, comparative studies are needed to clarify differences in quantitative indicators of pulpal blood flow among various diagnostic modalities, including LDF, PO, and TLP. Traumatized teeth often present a diagnostic “gray zone” immediately before becoming nonvital, where pulp vitality is difficult to determine and test results may be inconsistent. The findings of this study suggest that combining TLP with other diagnostic methods may help overcome individual limitations, thereby achieving higher overall diagnostic accuracy. To realize its full potential in clinical practice, prospective multicenter validation and device standardization will be required.

## Supplementary Information

Below is the link to the electronic supplementary material.

Supplementary Material 1

## Data Availability

The datasets used and/or analysed during the current study are not publicly available due to patient privacy and ethical restrictions but are available from the corresponding author on reasonable request.
