# Supplementary material for: Diagnostic accuracy of Transmitted-light plethysmography for the assessment of pulpal circulation in traumatized young permanent incisors
Source: Sci Rep. 2025 Nov 28;15:42579. doi: 10.1038/s41598-025-25063-8 (PMC12663260; doi:10.1038/s41598-025-25063-8)
Supplement: Supplementary file 1 — Supplementary Material 1 [file 41598_2025_25063_MOESM1_ESM.pdf]

**Supplementary Table:**

Distribution of root development stages according to the Moorrees classification

| Root development stage (Moorrees classification) | Group 1 Nontraumatized teeth (N = 37) | | Group 2 Traumatized vital teeth (N = 62) | | Group 3 Traumatized nonvital teeth (N = 32) | |
|---|---|---|---|---|---|---|
| | N | % | N | % | N | % |
| R1/2 - R3/4 | 12 | 32.4% | 32 | 51.6% | 18 | 56.3% |
| Rc | 9 | 24.3% | 17 | 27.4% | 6 | 18.8% |
| A1/2 | 4 | 10.8% | 5 | 8.1% | 4 | 12.5% |
| Ac | 12 | 32.4% | 8 | 12.9% | 4 | 12.5% |

R1/2–R3/4: Half to three-quarters root development with a wide-open apex.

Rc: Root length completed with an open apex (parallel canal wall).

A1/2: Completed root development with a half-closed apex and wide periodontal ligament (PDL).

Ac: Completed root development with a fully closed apex and normal PDL width.
